# Response to the Address of Welcome*Delivered July 20, 1898, at the meeting held at Atlanta, Ga.

**Published:** 1898-09

**Authors:** J. R. Shannon

**Affiliations:** Cabaniss, Ga.


### RESPONSE TO THE ADDRESS OF WELCOME.*

BY J. R. SHANNON, A.M., M.D., CABANISS, GA.

*Mr. President and Gentlemen of the Alumni Association of the Southern Medical College:*

It is with pleasure that I meet you to-day, and gladly do I give response in behalf of the association to the words of Dr. Parks of the local alumni.

While it is a pleasure to be with you to-day, there creeps over me a feeling of sadness that, since our last meeting, there are those who are absent forever, and I but voice the sentiment of all present when I say like Tennyson:

> " O, for the touch of a vanished hand,
> And the sound of a voice that is stilled."

While we give the kindly tear of sweet memory for our departed brethren, we give the hand of warm friendship and encouragement to the living.

It is meet and proper that we should assemble at this time and in the city of Atlanta. Surrounded as we are, and imbued with the sentiments that fill every mind and permeate every heart of every lover of his native country in this Southland of ours, we can not but feel a kindly interest in our fellow members and a worthy ambition to a higher attainment in our chosen profession. To-day the Confederate veterans from every nook and corner of Dixie land have met to greet each other and exchange friendly reminiscences of the past, to renew old friendships, and to pledge fidelity for the future. Thus we have met together to make afresh the memory of our college days and pledge fidelity to one another in the coming years, and offer our humble efforts upon the altar of medical science; and, by the exchange of ideas and experience, we hope to advance in some small degree the interests of each other and the benefit of the profession which we love and for which we live.

I have an abiding faith in the future of our profession in Georgia. With the new legislation now existing and the enactment of further laws for the protection and advancement of medical science, we see no reason why Georgia should not take her place among the foremost of the States in medical progress and medical enlightenment. To accomplish this most worthy end the means are at our command, if we will only take advantage of them. There is urgent need of further protection and encouragement by and through the law-abiding power of our country. If every physician will lend his hand and voice in advocating and encouraging the needful legislation, the time is not far distant when the wish will become a living reality. Then, and not till then, will Georgia

*Delivered July 20, 1898, at the meeting held at Atlanta, Ga.

have done her duty to the great army of physicians now within her boundaries. This needful legislation consists in better protection from quacks, a higher standard for admission to the colleges, and an increase in the length of sessions and in the number of years necessary for graduation. There is also a need for better collection laws for physicians. If every physician throughout the State would year in and year out do his duty, this much-needed reform would be accomplished.

Mr. President, as I stand here to-day, there comes to me in prophetic vision a beautiful scene of Atlanta at the end of another decade. I see her then as a city of vastly increased population and wealth, rivaling the largest city of the South. I see her educational institutions increasing in number and quality. I see her business enterprises enlarging and extending, and I see the strong arm of her social, commercial, intellectual, and moral power extending in every direction. Amid all this progress there will be no greater advancement in any line than in medical colleges. I see her increasing in number and in the standard of her medical institutions until her medical colleges will be to Georgia and the South what Bellevue is to New York and the North. May this vision of hope become a scene of reality. May she continue in the line of medical progress until her medical universities will have attained the high standard of the universities of Berlin and Vienna.

